# Treatment of imported severe malaria with artesunate instead of quinine - more evidence needed?

**DOI:** 10.1186/1475-2875-10-256

**Published:** 2011-09-07

**Authors:** Jakob P Cramer, Rogelio López-Vélez, Gerd D Burchard, Martin P Grobusch, Peter J de Vries

**Affiliations:** 1 Bernhard Nocht Clinic for Tropical Medicine, I. Department of Tropical Medicine, University Medical Center Hamburg-Eppendorf, Bernhard-Nocht-Strasse 74, 20359 Hamburg, Germany; 2 Department of Infectious Diseases, Tropical Medicine and Clinical Parasitology, Ramón y Cajal Hospital, University of Alcalá, Carretera de Colmenar Km 9,1, 28034 Madrid, Spain; 3 Division of Infectious Diseases, Tropical Medicine and AIDS, Department of Medicine, Amsterdam Medical Center, University of Amsterdam, PO Box 22600, 1100 DE Amsterdam, The Netherlands

## Abstract

Rapid and fast acting anti-malarials are essential to treat severe malaria. Quinine has been the only option for parenteral therapy until recently. While current evidence shows that intravenous artesunate is more effective than quinine in treating severe malaria in endemic countries, some questions remain regarding safety profiles and drug resistance. For imported severe malaria, additional unanswered questions are related to generalizability of the findings from endemic countries and to legal aspects, as there is no Good Manufacturing Practice-conform drug available yet. Here, the implications of existing evidence for the treatment of imported severe malaria are discussed.

## Background

Treatment of severe malaria requires prompt administration of safe and fast acting anti-malarials. While approximately 4% of all imported malaria cases progress to severe malaria, the overall mortality rate of imported *Plasmodium falciparum *malaria in Europe is 0,4% [1]. Until recently, quinine was the only option for parenteral therapy. Two large multinational randomized controlled unblinded trials (RCTs) have been conducted to compare intravenous quinine versus artesunate in malaria-endemic countries in Southeast Asia (SEAQUAMAT) and in sub-Saharan Africa (AQUAMAT) [2,3]. The evidence from these and some smaller trials supports the choice of artesunate over quinine in the treatment of severe malaria in endemic regions. But is this evidence sufficient to finally implement this anti-malarial in the treatment of imported severe malaria in non-endemic countries?

## Discussion

First, is it of concern that none of the existing trials has been performed as a double blind comparative study? This appears to be less important with respect to effectiveness as the end-point of the two large studies was mortality. However, some limitations regarding outcomes such as adverse events or (neurologic) sequelae remain and from a regulatory perspective this may well be of concern.

Second, can the existing evidence from endemic countries be generalized to imported severe malaria in industrialized, non-endemic, countries? Clinical manifestations, supportive intensive medical care and patient characteristics differ in endemic versus non-endemic countries. In particular, the following limitations have to be addressed: i) most evidence has been obtained from children while the great majority of patients with imported severe malaria are adults, ii) quinine monotherapy was used which does not completely reflect the often used combination of quinine with doxycycline or clindamycin in imported severe malaria, iii) the study population included a large variety of individuals from Southeast Asia, India and Africa. The characteristics of the study population with regard to ethnic, nutritional and general health aspects as well as to immunologic aspects (semi-immunity) may be different for patients with imported malaria (mostly caucasians, migrants). And lastly but possibly most importantly, iv), supportive care other than anti-malarial treatment may differ significantly in endemic settings versus hospitals in industrialized countries. Patients with imported malaria are monitored on intensive care units (ICU) and organ replacement therapy can be initiated promptly in case of renal or pulmonary insufficiency. Albeit speculative due to lacking evidence, these aspects appear to be major reasons why mortality is lower in imported versus endemic severe malaria.

The beneficial characteristics of artesunate are well known: it is a fast acting drug against several parasite stages including gametocytes [4,5], it has an apparently beneficial safety profile, and it reduces cytoadherence [6,7]. Better survival rates are observed among patients with severe malaria, particularly in those with high parasitaemia. This in turn may shorten the stay in the intensive care unit, which is beneficial for both the patient and the hospital. Shorter hospitalization also subsequently decreases the risk of nosocomial infections, which are quite frequently observed also in imported severe malaria [8]. Clinical benefits have been summarized recently in a meta-analysis [9].

Quinine, on the other hand, is rightfully claimed to be a drug with a narrow therapeutic window bearing the risk of a range of potential side effects like hypoglycaemia, agranulocytosis, bleeding disorders, cardiovascular effects and others which are particularly relevant for elderly patients [10]. While the administration of a bolus injection of artesunate is simple, the administration of quinine by continuous infusion is more complex. Furthermore, artesunate does not require dose reduction in renal insufficiency and there are no known drug-interactions while previous intake of mefloquine necessitates dose modifications of quinine. However, the adverse effects of quinine are well known and can be closely monitored under ICU-conditions.

Although artesunate has been used for the treatment of large numbers of patients, both in trials and under routine conditions, data on tolerability and safety on artesunate treatment are still limited for various reasons such as short hospitalization and follow-up time or incomplete laboratory measurements of adverse events in endemic countries. For example, cases of late-onset haemolysis have been reported recently occurring after artesunate treatment and this effect had not been documented in the published trials [11]. Furthermore, the relevance of additional safety aspects, including neurotoxicity, haematotoxicity/neutropaenia, embryotoxicity as well as allergic reactions, has not finally been clarified: toxic effects have been described in animals, but not in humans and they appear to be - in part - dose-dependent or may be related to delayed drug release after intramuscular application of arthemether/arteether but not to intravenous artesunate [12,13]. Not to forget that unanswered questions remain with respect to early evidence of drug resistance arising from Southeast Asia [14,15].

Finally, the currently available artesunate, produced by Guilin Pharmaceutical Company - the same product that was used in the -QUAMAT trials - is not being produced under conditions of full Good Manufacturing Practice (GMP). Guilin Pharmaceutical Company received pre-qualification by the World Health Organization in November 2010 but this is not the same as GMP certification by the European Medicines Agency (EMA) or US Food and Drug Agency. While there is no GMP-artesunate available in most countries and no licensed artesunate in any developed country, the availability of quinine is quite heterogeneous as well. In most industrialized countries, quinine is available through extemporaneous preparation. However, quinine is difficult to obtain or not available for intravenous administration in some European countries and the USA. Instead, a mixture of the four cinchona alkaloids (Quinimax^®^) rather than pure quinine salt is used in some European countries, which renders the therapy more heterogeneous with respect to pharmacokinetics and pharmacodynamics. While in the USA, a GMP-conform iv-artesunate has been made available but is not FDA-registered quinidine, which is more toxic than quinine still remains the first-line drug [16,17]. This background contributes to the fact that with the exception of the Netherlands and Australia, artesunate has not (yet) been incorporated as clear first-line drug in developed countries despite existing clear evidence on anti-malarial superiority of artesunate over quinine. In this context, it appears adequate to note that the manufacturing process and the quality of the quinine used in the -QUAMAT trials made by Indus Pharma, Karachi/Pakistan, may not be comparable with those of the artesunate from Guilin, which has been inspected to its compliance with WHO requirements for essential drugs.

## Conclusion

So, is additional evidence needed for replacing quinine as the drug of first choice in Europe and the USA? First, the anti-malarial superiority of artesunate over quinine has been established in endemic countries, but the effect could be smaller in developed countries. Second, additional data on drug safety are necessary, but can most likely only be obtained from surveys in developed countries. One may argue that none of the drugs currently used to treat imported severe malaria was ever studied by appropriate clinical trials and that evidence has always been extrapolated from paediatric treatment data in endemic areas. But this appears to be a flimsy argument in times of evidence-based treatment guidelines. However, a comparative study on imported severe malaria appears not to be feasible for several reasons: i) an unrealistically high number of subjects was needed if the outcome was mortality while other (combined) outcomes may not unequivocally answer relevant questions, ii) returning travellers are a very heterogeneous patient group requiring stratification (e.g. migrants visiting family and friends versus tourist travellers), and, probably most important, iii) many travel medicine experts would not be willing to randomize patients into artesunate versus quinine arms anymore given existing evidence and respective recommendations.

Despite the fact that artesunate is not a licensed drug in Europe it has been made available within compassionate use programmes via a few import companies (e.g. ACE Pharmaceuticals BV, the Netherlands, or Idis Pharma, UK) or it can be ordered directly from the manufacturer, Guilin Pharmaceutical Corporation in China. In the USA, the FDA has approved an investigational new drug protocol and GMP-conform intravenous artesunate has been made available via CDC/Walter Reed Army Institute of Research [6]. There are several pharmaceutical initiatives that aim for production under full GMP conditions (e.g. Sigma Tau, Italy) but it is still unclear when GMP-conform intravenous artesunate will become available in Europe. Several centers specialized in tropical/travel medicine have started to use intravenous artesunate and anecdotally confirm the clear shortening of the critical illness phase using artesunate in imported severe malaria supporting first data from the USA [18].

The most realistic approach is allowing the product to be used in a controlled fashion and to proactively document efficacy and toxicity. Since there are no established legal procedures for such a careful introduction of a still unregistered drug, clinicians and researchers have to take the lead. We have to organize ourselves and start collecting data on the use of artesunate for severe malaria in a concerted action. Properly collected post-introduction data are the most realistic alternative to the impossible option of prospectively collecting data in a comparative clinical trial. Such initiatives have already been put in place in Europe.

## Conflict of interest

The authors declare that they have no competing interests.
